# Improving Precision and Safety in Uterine Isthmocele Repair by Utilizing the KOH Cup and Firefly Guidance

**DOI:** 10.7759/cureus.79934

**Published:** 2025-03-02

**Authors:** Rooma Sinha, Bana Rupa, Amrutha Pentakota

**Affiliations:** 1 Department of Gynaecology Minimal Access Surgery, Apollo Health City, Hyderabad, IND

**Keywords:** da vinci, firefly, isthmocele, koh cup, robotic repair, uterine niche

## Abstract

Multiple surgical modalities are currently used for uterine isthmocele repair. Identifying defects intraoperatively remains challenging, and guidelines for optimal repair techniques are still evolving. Here, we present a case of safe and precise uterine isthmocele repair using the KOH Cup (Cooper Surgical Inc., Trumbull, USA) in combination with the Firefly technology of the Da Vinci Xi robotic system (Intuitive Surgical Inc., Sunnyvale, USA). A 30-year-old woman, para 1 living 1, with a history of previous cesarean section, presented with secondary infertility for the past seven years. A suspicion of a uterine isthmocele was revealed by ultrasonography. Magnetic resonance imaging (MRI) confirmed the diagnosis of a bulky uterus with a thinned-out anterior wall myometrium at the utero-cervical junction and an isthmocele of collection measuring 7.6 x 3.0 mm. The KOH Cup was used alongside Firefly-guided complete isthmocele repair and bladder adhesiolysis, with the patient being discharged within 24 hours. By using the KOH Cup with the Firefly imaging system, surgical accuracy can be improved through real-time feedback, making it a safe approach for uterine isthmocele repair. While the KOH Cup and Firefly combination appear to be a novel and promising approach, a direct comparison with traditional laparoscopic methods (without robotic guidance) is necessary to establish its clear advantages. Without supporting data or references, the claim of superiority remains speculative.

## Introduction

Cesarean sections (CS) currently represent 21.1% of global childbirths each year, and this is expected to increase to 28.5% (38 million cases) by 2030 [1]. In India, the prevalence of CS has risen from 17% to 21.5% over five years, exceeding the World Health Organization (WHO)'s recommended threshold of 15% [2]. The rising rate of CS is placing greater demands on surgeons to manage the associated complications. Isthmocele, also known as uterine niche, occurs in 20-70% of cases and is a complication of CS caused by defective closure or healing of the cesarean scar [3]. An isthmocele, often results from inadequate healing of the uterine wall following a cesarean incision, leading to a pouch-like myometrial defect [3]. Several factors contribute to its formation, including patient-related conditions such as genetic predisposition (e.g., Ehlers-Danlos syndrome, Marfan syndrome, Factor V Leiden mutation), gestational diabetes, hypertension, endometriosis, ectopic pregnancy, high body mass index (BMI), smoking, and uterine retroversion. Delivery-related factors also play a role, such as the type and level of the uterine incision, suturing techniques, pelvic adhesions, early cervical dilation, and ectopic pregnancy at the scar site. These elements collectively impact healing, increasing the risk of isthmocele formation [3]. 

Isthmocele is a potential complication of CS that often goes unnoticed, with infertility being its most significant consequence. It may contribute to infertility through several mechanisms. Local inflammation can impair endometrial receptivity and disrupt the uterine microbiota, hindering implantation [3]. A large isthmocele may obstruct sperm transport, while mucus or scar tissue can create a hostile uterine environment, reducing sperm survival. Fluid accumulation (hydrometra) within the defect may lower implantation rates due to embryotoxic effects, and excess iron from bleeding can further harm the embryo. Additionally, isthmocele can compromise uterine blood supply and nutrient availability, leading to reduced implantation rates and increased In vitro fertilization (IVF) complications [3]. Post-menstrual spotting, prolonged bleeding, and early cycle intermenstrual bleeding are all symptoms that are typical of a uterine isthmocele defect [4]. It has been reported that isthmocele often leads to infertility and reduces the success rate of IVF [3]. Retained blood in the isthmocele can lead to altered endometrial receptivity, endometrial thinning, or chronic endometritis, all of which can impair implantation. Chronic inflammation at the cesarean scar site may disrupt normal uterine function and embryo implantation. Furthermore, a meta-analysis found that women with an isthmocele had lower live birth rates. The adjusted odds ratio was 0.62 (95% CI, 0.53-0.72) versus those with prior CS without isthmocele and 0.55 (95% CI, 0.42-0.71) versus those with vaginal deliveries, indicating a 38% and 45% reduction, respectively [5]. Isthmocele can reduce IVF success rates by creating an unfavorable uterine environment. Local inflammation may impair endometrial receptivity and disrupt the microbiota, while fluid accumulation can hinder implantation due to embryotoxic effects. Additionally, isthmocele may disrupt blood supply and nutrient availability, further compromising implantation. Studies indicate lower implantation rates in women with larger isthmocele, highlighting its negative impact on IVF outcomes [3]. The condition is diagnosed through transvaginal ultrasound, but magnetic resonance imaging (MRI) can aid in evaluating its prognosis, particularly when there is abnormal uterine bleeding [6,7]. Various treatment modalities are currently in use, but identifying and accurately repairing the condition poses unique challenges, making management guidelines still a work in progress [6,8].

The management of isthmocele includes both medical and surgical options, depending on symptoms, fertility goals, and residual myometrial thickness (RMT). Medical treatments, such as oral contraceptives and progesterone-releasing intrauterine device (IUD), help regulate menstrual cycles and manage abnormal bleeding [3]. Surgical approaches vary based on defect characteristics and surgical expertise. A hysteroscopic approach is minimally invasive and preferred when RMT is at least 2.5-3.0 mm but is avoided in thinner defects due to potential complications [3,9]. The laparoscopic approach is recommended for RMT below 3 mm, allowing complete excision and suturing but is technically demanding [9]. The vaginal approach involves excision and double-layer closure but requires a longer hospital stay [6,9]. Robotic-assisted surgery offers enhanced precision and minimally invasive benefits, though more studies are needed to confirm its long-term effectiveness [8,9].

Firefly is an in-built feature of the Da Vinci Xi robotic system (Intuitive Surgical Inc., Sunnyvale, USA), that uses near-infrared rays, generated by indocyanine green dye or transillumination, to visualize and outline tissues, blood vessels, and lymphatics. Given the lack of clear consensus on the optimal treatment for uterine isthmocele repair, the authors utilized a cutting-edge surgical technique - the robotic approach equipped with Firefly mode. This method is safe and free of adverse effects. Transillumination using hysteroscopic light was employed to accurately locate the defect, while Firefly mode provided visual guidance, enabling the surgeon to perform a precise repair. The KOH Cup colpotomizer (Cooper Surgical Inc., Trumbull, USA) aids in the identification of the cervico-isthmic junction, and by leveraging Firefly technology, target tissues can be visualized more effectively, facilitating meticulous repair while minimizing tissue trauma.

Here, we present a case that describes the innovative use of the KOH Cup alongside the Firefly technology of the Da Vinci Xi robotic system for safe and complete isthmocele repair. The KOH Cup sits precisely in the vaginal cuff, just above the isthmus, helping to demarcate the defect's boundaries while also providing a visual guide for bladder dissection. In the discussed case, where the bladder was adherent, the KOH Cup facilitated a smoother and more precise dissection.

## Case presentation

**Figure 1:**
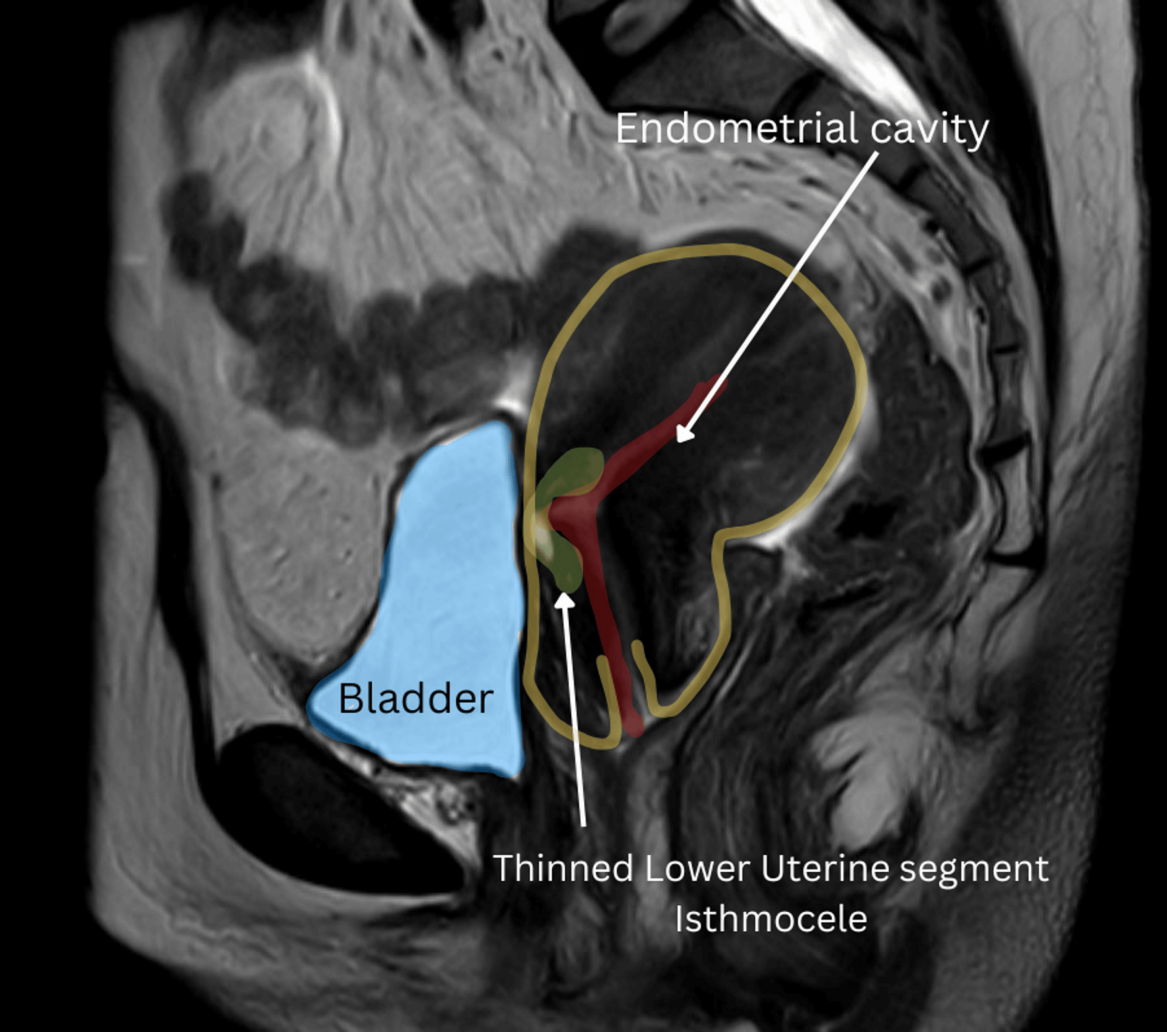
MRI visualization (T2-weighted sagittal section) of uterine isthmocele. The myometrial scar thickness is 3 mm. MRI: Magnetic resonance imaging

A 30-year-old woman, para 1 living 1, with a history of previous CS, presented to an infertility specialist with secondary infertility for the past seven years. The previous CS was performed due to fetal distress, with no reported history of significant postoperative complications. She had no other comorbidities. However, ultrasonography raised suspicion of a uterine isthmocele. She was referred to our center for surgical management. MRI suggested a bulky uterus with a thinned anterior wall myometrium at the utero-cervical junction, with a collection measuring 7.6 x 3.0 mm at the isthmocele (Figure 1).

**Figure 2:**
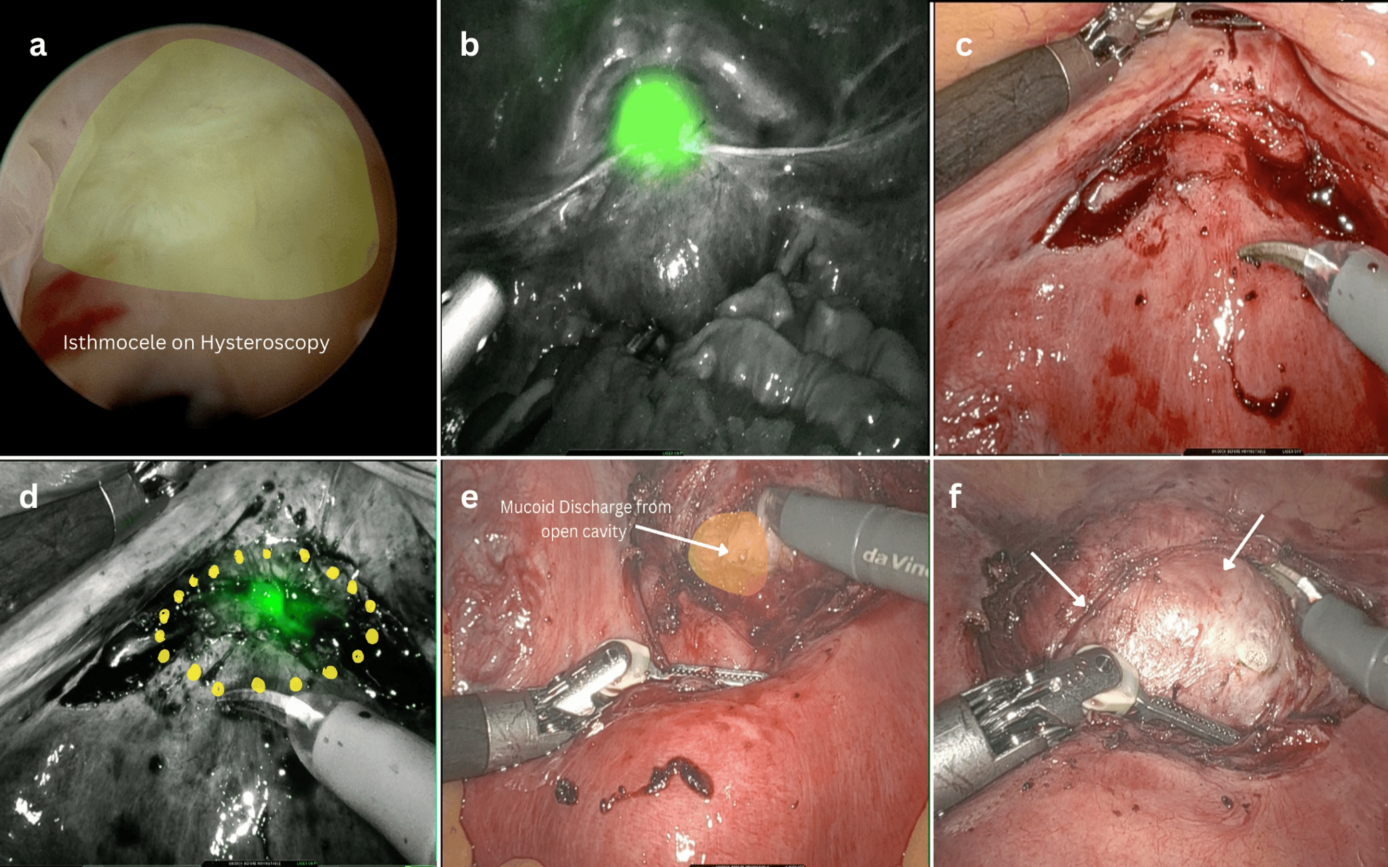
Surgical procedure. (a) Hysteroscopic identification of uterine isthmocele; (b) Initial detection of defect on the application of Firefly mode; (c) Dissection of adherent utero-vesicle peritoneum; (d) Precise marking of the defect using monopolar scissors on the application of Firefly; (e) Mucoid contents oozing out of the defect during dissection confirming the opening of the endometrial cavity; (f) KOH Cup rim aiding in bladder dissection and precise defect identification.

Typically, surgical intervention is recommended for a residual myometrial thickness of less than 3 mm. In our case, a thickness of 2.4 mm met the criteria for surgery. Following counseling and consent, the patient was administered general anesthesia. The surgical procedure began with hysteroscopy using normal saline as the distension medium to assess the extent and nature of the defect (Figure 2a). The Da Vinci Xi robot was docked after gaining abdominal access. Firefly mode was activated, allowing visualization of the defect’s initial location (Figure 2b). The adherent utero-vesical peritoneal fold was carefully dissected, and the bladder was dissected caudally (Figure 2c). By utilizing hysteroscopic transillumination along with Firefly guidance, the defect was identified and marked with monopolar curved scissors (Intuitive Surgical Inc., Sunnyvale, USA) (Figure 2d). The hysteroscope (Bettocchi, Karl Storz, Germany) was then withdrawn, and RUMI uterine manipulator (Cooper Surgical Inc., Trumbull, USA) with KOH Cup colpotomizer was inserted vaginally. Following the Firefly-guided markings, fibrotic tissue was excised with monopolar scissors (cut - 180w) until healthy myometrial tissue was visible. During this process, the mucoid material within the uterine isthmocele was drained (Figure 2e). With the KOH Cup-guided delineation of the cervico-isthmic junction and the uterine manipulator guiding the extent of depth, the entire defect was excised (Figure 2f).

**Figure 3:**
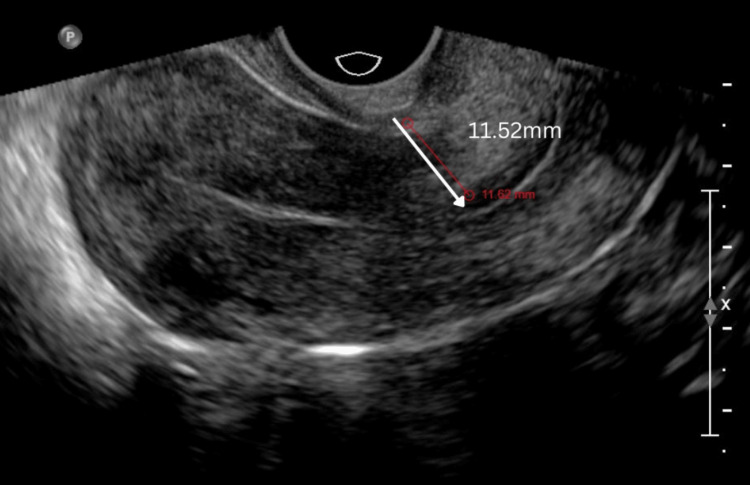
Postoperative ultrasonography with myometrial thickness of 11.52 mm.

A precise three-layer closure of the defect was performed using V-Loc barbed sutures (Covidien, Dublin, Ireland) under direct visualization. The procedure was well-tolerated by the patient, and she was discharged within 24 hours. A three-month follow-up of the case revealed a marked improvement in the residual myometrial thickness, increasing from 2.4 mm preoperatively to 11.52 mm postoperatively (Figure 3). The patient is currently under follow-up. Given the limited data in this field, the authors aimed to publish this novel technique to encourage global research and enhance understanding of its positive outcomes.

## Discussion

Infertility caused by an iatrogenic isthmocele can be a difficult experience. In our case report, the patient presented with seven years of secondary infertility. The previous conception was spontaneous, and the fertility parameters were normal at her presentation. A recent review proposed that isthmocele may cause infertility by creating a physical hindrance for the sperm to traverse and/or create an inflammatory, sperm-toxic environment due to accumulated menstrual blood and mucus [3]. In our case, we found mucoid debris visibly oozed out upon making the incision into the cavity. An image supporting this observation has been presented. A strengths, weaknesses, opportunities, and threats (SWOT) analysis by Dominguez et al. (2023) discussed that isthmocele correction can lead to spontaneous conception. The analysis showed that a transvaginal ultrasound was sufficient to diagnose the condition [6]. In the review by Baldini et al. (2024), it was found that MRI's accuracy in evaluating residual myometrial thickness is better [3]. Although it lacked statistical significance, the study summarized that MRI is critical for precise lesion mapping during surgical planning [3]. Furthermore, Zhang et al. (2023) concluded that MRI is essential for assessing the prognosis in cases of abnormal uterine bleeding [7]. For an accurate assessment of the defect of the isthmocele, we performed an MRI in the case reported in this paper. A 2024 review article found that MRI provided greater accuracy in evaluating residual myometrial thickness [3]. Acknowledging the paucity of consensus on treatment approach for this condition, Baldini et al. reviewed various surgical options for symptomatic patients, including hysteroscopic, laparoscopic, and robotic surgery. According to the authors, fluorescence guidance to delineate both the defect and the limits of the bladder adherent to the scar was an effective method to provide visual clues for appropriate defect closure and safe bladder dissection. The Da Vinci Xi robotic approach that was used in this case provided the Firefly with better maneuverability and precision [3]. Regarding robotic uterine isthmocele repair, evidence from a retrospective study of 33 cases (probably the largest existing data) suggests that robotic surgery is a viable option with significant improvement in myometrial thickness [8].

Similarly, our case report also demonstrated a notable improvement in the residual myometrial thickness on a follow-up ultrasound conducted four months later. In our study, we attempted fluorescein-guided correction using the robot's in-built Firefly technology by merely using hysteroscopic light transillumination, avoiding dye as a novel technique. The KOH Cup and Firefly technology offer a dye-free approach to uterine isthmocele repair with several advantages. Firefly’s near-infrared fluorescence enhances visualization by highlighting abnormal vasculature without dyes, which can obscure the field or cause allergic reactions. This technique reduces the risk of adverse responses associated with traditional dye-based methods, improving patient safety. Additionally, the combination of the KOH Cup for uterine manipulation and Firefly imaging enables precise defect localization and excision, preserving healthy myometrial tissue and supporting uterine integrity for future fertility. The KOH Cup of the RUMI uterine manipulation system fits well at the cervico-vaginal junction. The visual clue provided by the KOH Cup at the anterior cervico-vaginal junction resulted in better demarcation of boundaries and aided in the safe dissection of the bladder from the thinned-out lower uterine segment containing the isthmocele.

A recent review by Stavridis et al. (2024) highlighted surgical methods for uterine isthmocele correction, reporting one case of bladder injury and Clavien-Dindo II and IIIa complications following robotic correction [9]. In contrast to the findings, even though the bladder was densely adherent in our case, we performed a safe dissection of the bladder from the thinned-out uterine scar, guided by a KOH Cup and a Firefly. Additionally, the postoperative recovery was smooth, and the patient was discharged the next day.

## Conclusions

In the absence of guidelines for uterine isthmocele repair, a novel safe technique has been attempted. Precise and adequate repair of uterine isthmocele is important for scar integrity and improved reproductive outcomes. Latest advancements have been put to use, like the infrared camera with Firefly imaging to identify defects during surgery in real-time, enabling a robust repair. The KOH Cup, located at the thinned-out cesarean scar, provides a visual clue to the cervical uterine junction, which can prevent inadvertent cystotomy. Further, large studies in the field are required for complete understanding.
